# Health check revealed elevation of creatine kinase related to hypothyroidism

**DOI:** 10.1530/EDM-25-0123

**Published:** 2026-02-13

**Authors:** Xiaoqing Luo, Yamei Quan, Gaodi Luo

**Affiliations:** Health Examination Department of the First People’s Hospital of Shuangliu District, Chengdu, China

**Keywords:** thyroid, metabolism

## Abstract

**Summary:**

It is relatively rare to find a significant increase in creatine kinase (CK) related to hypothyroidism through a health examination. This case report describes an asymptomatic adult patient in whom an asymptomatic elevation of serum CK was discovered incidentally during a routine health check. The patient reported no significant muscle weakness, pain, or other classic symptoms of proximal myopathy or hypothyroidism. A comprehensive diagnostic workup was initiated to investigate the common causes of high CK level, including cardiac injury, strenuous exercise, statin use, and inflammatory myopathies, all of which were excluded. Thyroid function tests revealed severe primary hypothyroidism with significantly elevated thyroid-stimulating hormone (TSH; >100 mIU/L) and decreased free thyroxine levels. A diagnosis of hypothyroid myopathy was established. Upon initiation of levothyroxine replacement therapy, the patient’s thyroid function normalized, and this was followed by a complete normalization of the serum CK level on subsequent follow-up. This case highlights that hypothyroidism is an important, reversible, notable, and treatable cause of an asymptomatic unexplained CK elevation, particularly in the context of severe biochemical hypothyroidism. It underscores the critical importance of including thyroid function tests in the routine differential diagnosis of any unexplained high CK level, even in the absence of overt clinical myopathic symptoms or recognized signs of hypothyroidism. For clinicians and general practitioners interpreting routine health screening results, this case serves as a vital reminder that a simple thyroid function test can prevent unnecessary and costly investigations and lead to the correct diagnosis and effective treatment.

**Learning points:**

- In asymptomatic individuals undergoing routine health screening, an isolated elevation of creatine kinase (CK) may be an early biochemical indicator of underlying hypothyroidism, warranting simple thyroid function testing.
- This case underscores the public health value of routine biochemical panels that include CK, as they can identify individuals with subclinical endocrine dysfunction before the development of overt clinical disease.
- For primary care and preventive medicine physicians, integrating thyroid function tests into the initial workup for incidentally discovered hyperCKemia is a cost-effective strategy that can prevent unnecessary specialist referrals and invasive investigations.

## Background

An unexplained elevation of serum creatine kinase (CK), or hypercreatine kinaseemia, is a common incidental finding on routine health screening panels in China. The differential diagnosis is broad, encompassing cardiac injury, muscular dystrophies, inflammatory myopathies, viral infections, medication side effects (e.g. statins), and strenuous exercise. Among the endocrine causes, hypothyroidism is a well-established but frequently overlooked etiology. Hypothyroid myopathy, which is usually a late manifestation of severe disease, typically presents with symptoms such as proximal muscle weakness, stiffness, and delayed deep tendon reflexes. However, in its early stages or in the context of health screening, a markedly elevated CK level can precede the full development of symptomatic myopathy. In such scenarios, non-specific symptoms of hypothyroidism (e.g. fatigue and mild edema) may be subtle, overlooked, or absent.

This case is significant because it describes a common clinical scenario in preventive medicine – the management of an incidental finding of significant hyperCKemia – that unveiled a readily treatable endocrine disorder, severe hypothyroidism. It underscores a critical and underappreciated association in primary care and health screening practice. While hypothyroidism is common, its presentation with an isolated, marked CK elevation in the absence of recognized overt clinical symptoms is a well-documented but often forgotten diagnostic possibility, making it a challenging scenario for clinicians who first review routine blood results. The case highlights a crucial diagnostic pitfall: the failure to include thyroid function tests in the initial workup of unexplained hyperCKemia, which can lead to unnecessary, costly, and invasive investigations. By demonstrating a rapid normalization of CK levels with levothyroxine therapy, this case reinforces the link between thyroid hormone deficiency and muscle metabolism. Therefore, this report is highly relevant as it serves as an important reminder to clinicians, particularly in health checkup settings, of a simple, cost-effective diagnostic step that can prevent misdiagnosis and ensure timely treatment.

## Case presentation

A 47-year-old woman presented for a routine health checkup at the First People’s Hospital of Shuangliu District, Chengdu, on August 30, 2024. She reported no personal history of major systemic diseases, including diabetes, hypertension, coronary heart disease, or thyroid disorders. Her family history was significant for hypertension. She denied any history of trauma, surgery, or drug allergies. She described normal growth and development. Her lifestyle habits included no tobacco or alcohol use and no regular strenuous exercise. She reported average sleep quality, denied any psychiatric illness, and stated she undergoes a physical examination annually.

## Investigation

Initial laboratory testing from the routine health checkup revealed a significantly elevated serum CK level of 1,697.5 U/L (reference range (RR): 40–200). Other muscle enzymes were also elevated – lactate dehydrogenase (LDH): 329.4 U/L (RR: 120–250), α-hydroxybutyrate dehydrogenase (α-HBDH): 238.2 U/L (RR: 5.9–126.4), and aspartate aminotransferase (AST): 57.3 U/L (RR: 13–35).

Recognizing this as a critical abnormal finding, the attending physician promptly contacted the patient. Upon detailed inquiry, the patient reported generally feeling well but acknowledged mild, non-specific symptoms that had been overlooked, including intermittent facial puffiness and persistent, mild fatigue. She specifically denied chest pain, palpitations, muscle weakness, myalgia, or cramps. There was no history of statin use or recent vigorous exercise.

The patient was urgently recalled for a comprehensive clinical assessment. Her vital signs were stable: blood pressure 109/64 mmHg; heart rate 76 bpm. Her body mass index was 23.2 kg/m^2^. Physical examination revealed no cardiomegaly or abnormal heart sounds. The thyroid gland was not palpable or visibly enlarged. A focused neurological and musculoskeletal examination was performed, which documented normal bulk, tone, and strength (5/5 in all major muscle groups) in all four limbs. An electrocardiogram was not obtained during this initial outpatient evaluation.

Supplementary laboratory investigations were obtained: complete blood count: hemoglobin 105 g/L (RR: 115–150); urinalysis: protein negative, occult blood 1+; and renal function: serum creatinine 80.2 μmol/L (RR: 41–73).

Thyroid function tests were as follows (see Table 1 for full details) – thyroid-stimulating hormone (TSH): > 100.00 mIU/L (RR: 0.27–4.20), free thyroxine (FT4): < 0.50 pmol/L (RR: 12.00–22.00), and free triiodothyronine (FT3): < 0.60 pmol/L (RR: 3.10–6.80). Thyroid autoantibodies were markedly elevated – anti-thyroglobulin antibody (TgAb): > 4,000.00 IU/mL (RR: ≤115.00) and thyroid peroxidase antibody (TPOAb): 413.00 IU/mL (RR: ≤ 34.00). Thyroid ultrasonography showed a slightly small gland with heterogeneous echotexture and bilateral hypoechoic nodules (TI-RADS 3).

**Table 1:** Relevant test indicators during the physical examination.

Parameter	Value	Reference range
CK, U/L	1,697.5	40–200
LDH, U/L	329.4	120–250
α-HBDH, U/L	238.2	5.9–126.4
AST, U/L	57.3	13–35
Hb, g/L	105	115–150
RBC, 10^12^/L	3.25	3.8–5.1
Creatine, μmol/L	80.2	41–73
TT3, nmol/L	<0.30	1.30–3.10
FT3, pmol/L	<0.60	3.10–6.80
TT4, nmol/L	<5.40	66.00–181.00
FT4, pmol/L	<0.50	12.00–22.00
TSH, mIU/L	>100.00	0.27–4.20
TgAb, IU/mL	>4,000.00	≤115.00
TPOAb, IU/mL	413.00	≤34.00
TRAb, IU/L	12.10	0–1.75

Given the combination of profound biochemical hypothyroidism and markedly elevated CK in the absence of common cardiac, exertional, or drug-related causes, hypothyroid myopathy was strongly suspected as the underlying diagnosis. The patient was counseled on the potential risks and was urgently referred to the endocrinology department for further evaluation and initiation of hormone replacement therapy.

## Treatment

After admission, an electrocardiogram showed sinus rhythm with inverted T waves and a low voltage in the limb leads. Cardiac color Doppler ultrasound showed no significant abnormalities in cardiac structure or blood flow, and left ventricular systolic function was normal. High-sensitivity troponin I was within the normal range. Serology for hepatitis C core antigen was negative. Electromyography and muscle biopsy were not performed. The final diagnosis was primary hypothyroidism (presumably autoimmune, given the antibody profile). Treatment was initiated with levothyroxine replacement therapy.

## Outcome and follow-up

After initiation of levothyroxine, the patient’s facial edema subsided and she reported a marked improvement in energy levels and overall well-being. After discharge, the examinee followed the doctor’s advice for regular follow-up visits at the outpatient department. On September 20, 2024, thyroid function was rechecked at the endocrinology outpatient department – TSH: > 100.00 mIU/L, FT3: 1.82 pmol/L, and FT4: 7.00 pmol/L, and the CK level dropped to 502.8 U/L. In November 2024, repeat thyroid function tests showed the following – TSH: 2.200 mIU/L, FT3: 4.33 pmol/L, and FT4: 21.60 pmol/L, all within the normal range (Table 2). On August 18, 2025, a follow-up routine physical examination was conducted. The myocardial enzyme spectrum was rechecked – CK: 51.7 U/L, LDH: 136.3 U/L, α-HBDH dehydrogenase: 92.2 U/L, and AST: 22.2 U/L, all within the normal range (Table 3). The normalization of thyroid function paralleled the complete resolution of the elevated CK, confirming the diagnosis of hypothyroidism-related hyperCKemia.

**Table 2:** Value of thyroid function and myocardial enzyme levels after thyroid hormone replacement therapy.

Parameter	After treatment			Reference range
	At 1 month	At 3 months	At 6 months	
FT3, pmol/L	1.82	4.33	4.46	3.10–6.80
FT4, pmol/L	7.00	21.60	21.20	12.00–22.00
TSH, mIU/L	>100.00	2.200	3.710	0.27–4.20
CK, U/L	502.8	-	-	40–200

**Table 3:** Levels of myocardial enzymes after thyroid hormone replacement therapy.

Parameter	Value	Reference range
CK, U/L	51.7	40–200
LDH, U/L	136.3	120–250
α-HBDH, U/L	92.2	5.9–126.4
AST, U/L	22.2	13–35

## Discussion

An unexplained elevation of serum CK (hyperCKemia) has a broad differential diagnosis, including cardiac injury, muscular dystrophies, inflammatory myopathies, viral infections, medication side effects (e.g. statins and antipsychotics), and strenuous exercise (1, 2). Among the rarer endocrine causes, hypothyroidism is a well-recognized but often overlooked entity (3). When a significantly elevated CK is discovered during a routine health check, a structured assessment is imperative. This includes a detailed history to exclude common causes. If these common causes are not evident, thyroid function testing should be considered in the initial evaluation (4).

Hypothyroidism can cause elevated serum CK, primarily through altered muscle metabolism (5). The classic symptomatic hypothyroid myopathy, characterized by proximal muscle weakness, is a late manifestation (3). However, an asymptomatic or mild CK elevation can be an early biochemical sign (5). The degree of elevation is typically mild to moderate (3, 5). The patient described herein presented with profound hypothyroidism and a markedly elevated CK, which normalized rapidly with levothyroxine, supporting the causality (5).

The key implication of this case is for health screening practice: an isolated, unexplained hyperCKemia warrants consideration of hypothyroidism in the differential diagnosis (4). This is not to suggest CK as a screening tool for hypothyroidism, but rather that checking TSH in this specific context is a simple and cost-effective step that can prevent unnecessary investigations (4, 6).

In conclusion, this report highlights that in the context of a routine health check, marked hyperCKemia can be the sole initial pointer to severe hypothyroidism. It underscores the value of including thyroid function tests in the initial workup of an unexplained CK elevation as a resource-efficient diagnostic strategy (4, 6).

## Declaration of interest

The authors declare that there is no conflict of interest that could be perceived as prejudicing the impartiality of the work reported.

## Funding

This work did not receive any specific grant from any funding agency in the public, commercial, or not-for-profit sector.

## Patient consent

Written informed consent was obtained from our patient for the publication of this case report.

## Author contribution statement

XL and YQ contributed to the conception and design of this manuscript. The content of this article has been approved and authorized by the attending physician of the patient.
